# Exercise effects on muscle quality in older adults: a systematic review and meta-analysis

**DOI:** 10.1038/s41598-021-00600-3

**Published:** 2021-10-26

**Authors:** Régis Radaelli, Dennis R. Taaffe, Robert U. Newton, Daniel. A. Galvão, Pedro Lopez

**Affiliations:** 1 Neuromuscular Research Lab, Faculty of Human Kinetics, University of Lisbon, Cruz Quebrada Dafundo, Portugal; 2 Exercise Medicine Research Institute, Edith Cowan University, 270 Joondalup Drive, Joondalup, WA 6027 Australia; 3 School of Medical and Health Sciences, Edith Cowan University, Joondalup, WA Australia; 4 School of Human Movement and Nutrition Sciences, University of Queensland, St. Lucia, QLD Australia

**Keywords:** Musculoskeletal system, Lifestyle modification

## Abstract

To systematically review and analyse the effects of exercise on morphological and neuromuscular muscle quality (MQ) outcomes in older adults and assess a range of possible moderators that may affect the impact of exercise on MQ outcomes. Using PRISMA guidelines, randomised controlled trials were searched in CINAHL, EMBASE, LILACS, PubMed, SciELO, Web of Science, MedNar, OpenGrey and OpenThesis databases. Eligible trials examined the effects of exercise interventions on morphological and neuromuscular MQ in older adults (≥ 60 years). Twenty-one trials (n = 973 participants) were included. Exercise significantly improved morphological MQ (effect size (ES) = 0.32, 95% CI 0.13–0.51, P < 0.001) with significant results maintained for studies assessing muscle density and intermuscular adipose tissue (ES = 0.45–0.52, P < 0.05). For neuromuscular MQ, exercise provided significant positive effects (ES = 0.49, 95% CI 0.29–0.69, P < 0.001) but only maintained for physically healthy participants (ES = 0.43, P < 0.001), resistance exercise interventions (ES = 0.64, P < 0.001), or studies assessing 1-RM or knee extensor isokinetic muscle strength relative to leg lean mass (ES = 0.48–0.62, P = 0.001). Associations between exercise duration and changes in MQ measures were not observed (P > 0.05). Supervised exercise interventions significantly improved different measures of MQ regardless of exercise duration, although these effects were small-to-moderate and not supported across all population-, exercise-, and methods-related features.

## Introduction

Exercise is increasingly acknowledged for the numerous benefits for the musculoskeletal system such as increases in muscle function, quantity and quality in a wide range of healthy and clinical populations^1,2^. In older adults, for example, the utilization of exercise interventions has been considered crucial to mitigate muscle function declines and impaired mobility^3,4^, as well as reduce the risk of metabolic abnormalities^5^ and attenuate increases in intramuscular and intermuscular fat^6,7^. Muscle quality (MQ) is attracting research and clinical interest, providing information on lower limb muscle morphology and function as well as insight into the deterioration of muscle tissue over the lifespan and potential interventions to attenuate such consequences in older adults^8^.

The term muscle quality generally refers to two specific measures: morphological and neuromuscular MQ. Morphological MQ refers to the intermuscular and intramuscular adipose and fibrous tissue, effectively the amount of non-contractile tissue expressed in absolute terms and relative to total muscle size^8,9^. This measure is derived from imaging assessment (e.g., magnetic resonance imaging (MRI)^10^, peripheral quantitative computed tomography (pQCT) or computed tomography (CT)^11^, or ultrasound imaging (US)^12^). Neuromuscular MQ is the force produced per unit of muscle mass and is assessed by the ratio between a wide range of muscle strength (e.g., one-repetition maximum (1-RM), isometric and isokinetic) and muscle size assessments (e.g., muscle thickness, cross-sectional area, muscle volume, and lean mass)^13^. Both morphological and neuromuscular MQ maintenance or improvement are deemed important for older adults in order to preserve or enhance physical function and metabolic health^8,9^. Although it has been proposed that specific exercises such as resistance training (i.e., anabolic exercise; performing sets of repeated movements against a resistance with prominent effects observed on the musculoskeletal and neural systems), aerobic exercise (i.e., activity involving large muscle groups and performed in a continuous or intermittent fashion over an extended period of time, such as cycling, swimming, jogging or running with prominent effects observed on cardiorespiratory fitness and blood lipid profiles), or the combination (i.e., combined resistance and aerobic exercise, or concurrent training) may improve MQ measures through molecular pathways and anti-inflammatory effects^8,14,15^, or by enhancing muscle strength along with muscle mass^16^, this does not appear to consistently occur across studies involving older adults. For example, while exercise promotes significant enhancement of morphological MQ outcomes as reported in previous studies undertaken in frail and older adults with moderate limited functional capacity^17,18^, the same effect has not been observed in physically healthy older adults^19,20^, suggesting that older adults presenting at higher risk for disabilities may have greater capacity to adapt to exercise training with improvements in MQ. Moreover, although muscle strength is augmented to a greater extent and faster than muscle size resulting in increased neuromuscular MQ^16,18,21^, this is also conflicting with previous studies undertaking different assessment methods or assessing different muscles presenting no meaningful change following resistance exercise ^19,20^. Thus, it is unclear whether specific study characteristics such as the population included, assessment techniques, or even different intervention characteristics (e.g., exercise mode, alone or combined with nutrition programs) are influencing the magnitude of exercise effects on morphological and neuromuscular MQ features. Furthermore, despite a previous meta-analysis examining exercise effects on intermuscular adipose tissue and muscle density in adults with different metabolic disorders^22^, the lack of specific analyses involving older adults preclude determining the efficacy of exercise strategies in this population.

Given the abovementioned conflicting observations of exercise effects on MQ in older adults, the aim of this study was to systematically review and analyse the effects of exercise on morphological and neuromuscular MQ of the lower limb derived from MRI, CT and US imaging assessment, and ratios of muscle strength per muscle size, respectively, in older adults. In addition, a range of possible population-, exercise-, and methods-related variables that may affect the impact of exercise on MQ outcomes were examined by subgroup and meta-regression analyses.

## Results

### Studies included

**Figure 1:**
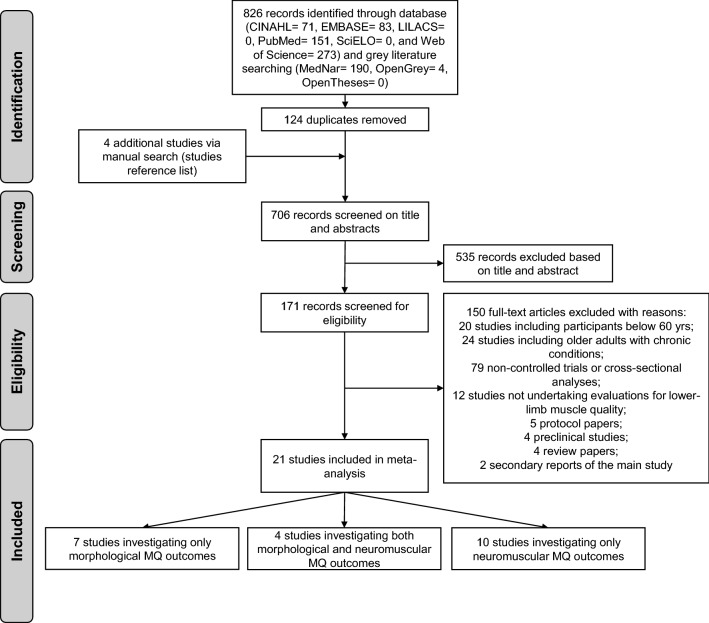
Flow chart of study selection process.

Eight-hundred twenty-six studies were retrieved from our search, with 706 potential records retained for screening after duplicate removals. Of these, 535 studies were excluded due to their irrelevance to the research question and 171 were deemed eligible and undertaken for full-text assessment (Fig. 1). A total of 21 randomised controlled trials (See in Table S1, SDC 2, Study characteristics: experimental design and sample size, exercise prescription and outcomes assessed) were included in the primary analysis^17–21,23–38^; 7 studies investigating only morphological MQ outcomes^17,23,25,29,30,36,38^, 10 studies investigating only neuromuscular MQ outcomes^21,26–28,31–35,37^, and 4 studies investigating both MQ measures^18–20,24^.

### Participants and study design characteristics

A total of 973 older adults (women, n = 651; men, n = 284; not reported, n = 38) with a median age of 70.3 years (interquartile range (IQR): 67.3–74.8) and BMI of 27.5 kg m^−2^ (IQR: 25.5–28.4) participated in the included studies. Most studies (n = 15) included physically healthy older adults^19–21,23,25,26,28–31,33–35,37,38^. All exercise interventions were supervised and included predominantly resistance exercise (13 of 21 studies)^19–21,23–25,28,31–34,36,37^, followed by multimodal exercise programs^17,18,38^ and aerobic exercise (3 of 21 studies)^23,30,35^, combined resistance and aerobic exercise (2 of 21 studies)^26,29^, and aquatic resistance exercise (1 of 21 studies)^25^. In addition, four studies prescribed exercise (i.e., combined resistance and aerobic exercise, resistance exercise, or aerobic exercise) allied with nutrition interventions such as caloric restriction, amino acids or protein supplementation^27,33,35,36^. Studies were designed to compare the exercise interventions versus non-active groups (12 of 21 studies)^20,21,23,25,26,28–30,32,34,36,37^, stretching or stretching and walking exercise groups (3 of 21 studies)^17,19,31^, health education classes (2 of 21 studies)^18,27^, and dietary education classes^24^, nutrition placebo^35^, walking^38^ or cognitive exercises^33^. Six studies compared multiple exercise interventions^23,25,29,33,35,36^. For the assessment of morphological MQ, 5 studies had undertaken CT^17,18,23,24,30^ or US imaging assessments^19,20,28,29,36^, and 2 studies had used the images derived from pQCT^25,38^. Seven studies had assessed the neuromuscular MQ by isokinetic muscle strength^18,19,26,27,31,33,35^ relative to DXA^27,31,33,35^, CT^18,26^ or US^19^, 4 studies assessed isometric muscle strength^19,26,32,34^ relative to CT^26^, DXA^32^, US^19^ or bioelectrical impedance analysis^34^, and 5 studies assessed 1-RM muscle strength^20,21,24,28,37^ relative to DXA^20,28,37^, CT^24^ or US^21^.

Regarding exercise prescription characteristics, the mean intervention duration was 18.3 ± 11.6 weeks (ranging from 6 to 48 weeks) with either 1^30,31^, 2^17,19–21,23,25,28,29,33,34,36,38^ or 3 sessions per week^24,26,27,32,35,37^, while the exercise frequency ranged from 1 to 3 sessions per week in one study^18^. Information about exercise volume^17,19–21,23,24,26,28–32,34,35,37^ and intensity^17,19–21,23–26,28,29,31,32,34,35,37^ were both reported by 15 studies. Adverse events related to the exercise programs were identified in 4 studies^23,24,30,31^, whereas 7 studies reported no adverse events throughout the exercise program period^17,18,25,32,33,35,36^. Ten studies did not report information about adverse events^19–21,26–29,34,37,38^.

### Risk of bias assessment

For the morphological MQ outcome assessment, 54.5% of the studies had *some concerns* (6 of 11 studies^17,18,23–25,36^), whereas 27.3% had a *high risk* (3 of 11 studies^20,29,30^) in the overall risk of bias assessment (Table 1). The high risk of bias in morphological MQ was in the *randomisation process* as 1 study did not report concealment allocation and present baseline differences between groups in the outcome assessed^20^, in *missing outcome data* as 1 study did not present the outcome of interest for all or nearly all participants^30^, and in the *measurement of the outcome* as outcome assessors were aware of the intervention received by the participants^20,29^. Regarding the studies presenting *some concerns*, these were in the *randomisation process* as studies did not report concealment allocation^18,23–25,29,30,36^ or presented baseline differences between groups in the outcome assessed^17^.

**Table 1:** Risk of bias of included studies.

Outcome	Randomisation process	Deviation from intended interventions	Missing outcome data	Measurement of the outcome	Selection of the reported result	Overall bias
**Morphological MQ, n = 11**^**a**^						
Low risk	2 (18.2%)	11 (100%)	10 (90.9%)	9 (81.8%)	11 (100%)	2 (18.2%)
Some concerns	8 (72.7%)	0	0	0	0	6 (54.5%)
High risk	1 (9.1%)	0	1 (9.1%)	2 (18.2%)	0	3 (27.3%)
**Neuromuscular MQ, n = 14**^**b**^						
Low risk	1 (7.1%)	14 (100%)	13 (92.9%)	8 (57.1%)	14 (100%)	1 (7.1%)
Some concerns	8 (57.1%)	0	0	0	0	5 (35.7%)
High risk	5 (35.7%)	0	1 (7.1%)	6 (42.9%)	0	8 (57.1%)

MQ, muscle quality; n, number of studies.

^a^Intention-to-treat analyses, n = 8 and per-protocol analyses, n = 3.

^b^Intention-to-treat analyses, n = 5 and per-protocol analyses, n = 9.

In the neuromuscular MQ overall risk of bias assessment, 57.1% of the studies had *high risk*^20,24,27,28,31,33,35,37^, whereas 35.7% had *some concerns*^18,21,26,32,34^ (Table 2). The high risk of bias was due to the *randomisation process* as studies did not report concealment allocation and present baseline differences between groups in the outcome assessed^20,27,28,33,35^, in the *missing outcome data* as 1 study did not present the outcome of interest for all or nearly all participants^33^, and in the *measurement of the outcome* as outcome assessors were aware of the intervention received by the participants^20,24,27,28,31,37^. Regarding the studies presenting *some concerns*, these were in the *randomisation process* as studies did not report concealment allocation^18,21,24,26,34,37^ or present baseline differences between groups in the outcome assessed^31,32^. The individual risk of bias assessment is presented in SDC 3 Figure S1A and S1B (see in SDC 3, Individual risk of bias assessment at outcome level).

**Table 2:** Overall and subgroup exercise effects on MQ derived from morphological outcomes in older adults.

Main effects	n	Sample size	SMD (95% CI)	I^2^	P-value
Overall effect	11	472	0.23 (− 0.01 to 0.48)	52%	0.062
Without outlier	10	387	0.32 (0.13 to 0.51)	3%	< 0.001
**Risk of bias**					
Low risk of bias	2	109	− 0.26 (− 0.57 to 0.05)	3%	0.104
Some concerns or high risk of bias	9	363	0.33 (0.13 to 0.05)	11%	0.001
**Population**					
Physically healthy	7	295	0.08 (− 0.20 to 0.36)	38%	0.586
Sarcopenia/dynapenia^†^	1	84	0.23 (− 0.22 to 0.69)	–	–
Moderate limited functional capacity^†^	1	42	0.40 (− 0.06 to 0.86)	–	–
Overweight/obese^†^	1	27	1.01 (0.40 to 1.62)	–	–
Frail^†^	1	24	0.20 (− 0.40 to 0.80)	–	–
**Intervention delivery**					
Supervised	11	472	0.23 (− 0.01 to 0.48)	52%	0.062
Unsupervised	-	-	−	–	–
**Intervention modality**					
Resistance exercise	6	196	0.34 (− 0.13 to 0.81)	65%	0.161
Resistance exercise + nutrition^†^	1	56	0.55 (0.01 to 1.09)	–	–
Aerobic exercise	2	55	0.18 (− 0.27 to 0.62)	0%	0.443
Combined resistance and aerobic exercise^†^	1	36	0.36 (− 0.32 to 1.05)	–	–
Multimodal exercise program	3	151	0.06 (− 0.43 to 0.55)	72%	0.812
Aquatic resistance exercise^†^	1	36	0.25 (− 0.40 to 0.90)	–	–
**Assessment methods—thigh**					
Muscle density^†^	1	42	0.11 (− 0.49 to 0.72)	–	–
High muscle density	4	122	0.52 (0.05 to 0.99)	34%	0.030
Low muscle density	3	80	0.34 (− 0.16 to 0.83)	19%	0.186
Intermuscular adipose tissue	3	98	0.45 (0.05 to 0.86)	0%	0.027
Echo intensity	3	146	0.21 (− 0.12 to 0.55)	0%	0.220
**Assessment methods—calf**					
Muscle density	2	138	− 0.18 (− 0.61 to 0.23)	32%	0.377
Intermuscular adipose tissue^†^	1	85	− 0.28 (− 0.71 to 0.14)	–	–
Echo intensity^†^	1	24	0.12 (− 0.68 to 0.92)	–	–

^†^Insufficient data for analysis; I^2^, indicator of between-study heterogeneity; n, number of studies; SMD, standardised mean difference.

### Exercise effects on morphological muscle quality

**Figure 2:**
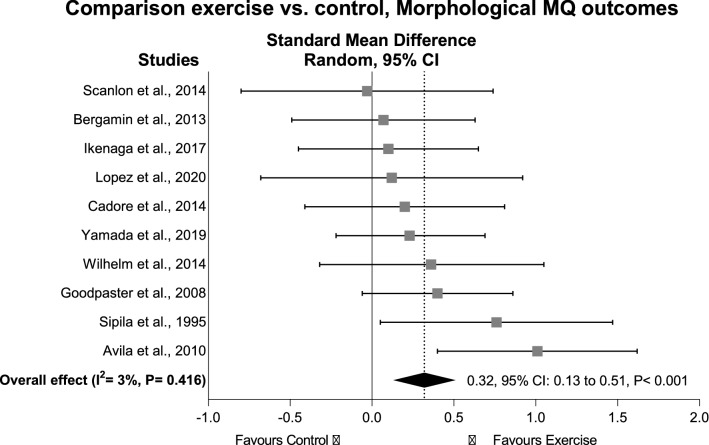
Standardised mean difference effects of exercise compared with control on morphological muscle quality outcomes in older adults. Overall analysis conducted with a random-effects model. Diamond represents pooled standardised mean difference estimate of random-effects meta-analysis; *I*^2^ represents the heterogeneity test; *MQ*, muscle quality; squares represent study-specific estimates.

Exercise resulted in a significant positive ES of 0.32 (95% CI 0.13–0.51, P < 0.001) in morphological MQ outcomes in 231 older adults who undertook supervised exercise interventions compared to 156 older adults in control groups (Fig. 2). The study of Minett et al.^38^ was considered an outlier and removed from the overall effect analysis (Table 2). The heterogeneity was I^2^ = 3% with no presence of publication bias (τ = 0.19, P = 0.918; see in SDC 4 Figure S2A, Contour-enhanced funnel plot). For studies assessing thigh high muscle density and intermuscular adipose tissue, there was a significant ES of 0.52 (95% CI 0.05–0.99, P = 0.030) and 0.45 (95% CI 0.05–0.86, P = 0.027), respectively, for exercise compared to control. However, the results were not maintained across the subgroup analyses regarding *low risk of bias* (ES = − 0.26, P = 0.104), population (physically healthy: ES = 0.08, P = 0.586) and intervention modalities (resistance exercise: ES = 0.34, P = 0.161; aerobic exercise: ES = 0.18, P = 0.443; and multimodal exercise program: ES = 0.06, P = 0.812). Furthermore, exercise effects were not significant for low muscle density (ES = 0.34, P = 0.186) and echo intensity (ES = 0.21, P = 0.220) of the thigh, and calf muscle density (ES = − 0.18, P = 0.377). Additional subgroup analyses for different study populations, intervention delivery and modality, and assessment methods were not undertaken given the small number of studies included (< 2). No significant association was observed between intervention duration and effects on morphological MQ outcomes (ranging from 6 to 48 weeks; β = 0.01, 95% CI − 0.02 to 0.03, P = 0.649), while associations of exercise prescribed volume and peak intensity with exercise effects were not undertaken given the high heterogeneity in the reporting of these exercise components.

### Exercise effects on neuromuscular muscle quality

**Figure 3:**
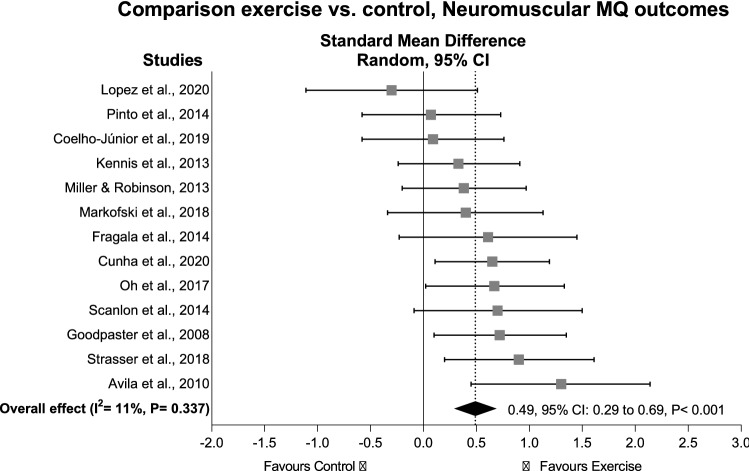
Standardised mean difference effects of exercise compared with control on neuromuscular muscle quality outcomes in older adults. Overall analysis conducted with a random-effects model. Diamond represents pooled standardised mean difference estimate of random-effects meta-analysis; *I*^2^ represents the heterogeneity test; *MQ*, muscle quality; squares represent study-specific estimates.

For the neuromuscular MQ outcomes, there was a significant ES of 0.49 (95% CI 0.29–0.69) (P < 0.001) in 271 older adults who undertook supervised exercise compared to 211 older adults in the control groups (Fig. 3). The study of Liao et al.^32^ was considered an outlier and removed from the overall effect analysis (Table 3). The heterogeneity was I^2^ = 11% with no publication bias identified (τ = 0.76, P = 0.730; see in SDC 4 Figure S2B, Contour-enhanced funnel plot). The results were maintained in subgroup analyses involving physically healthy older adults (ES = 0.43, 95% CI 0.21–0.64, P < 0.001), studies undertaking resistance exercise interventions (ES = 0.64, 95% CI 0.27–1.01, P < 0.001), and studies assessing 1-RM of the knee extensors relative to leg lean mass by DXA (ES = 0.62, 95% CI 0.26–1.05, P = 0.001) or knee extensor isokinetic muscle strength relative to leg lean mass by DXA (ES = 0.48, 95% CI 0.19–0.78, P = 0.001), while it was not significant in overweight/obese older adults (ES = 0.79, P = 0.082) or in studies assessing knee extensor isokinetic muscle strength relative to muscle volume by CT (ES = 0.42, P = 0.155). Additional subgroup analyses for risk of bias, different study populations, intervention delivery and modality, and assessment methods were not undertaken given the small number of studies included (< 2). No significant association was observed between intervention duration and effects on neuromuscular MQ outcomes (ranging from 6 to 48 weeks; β = − 0.00, 95% CI − 0.02 to 0.02, P = 0.868), while further analysis involving exercise components were not undertaken given the high heterogeneity in the reporting of these exercise components.

**Table 3:** Overall and subgroup exercise effects on MQ derived from neuromuscular outcomes in older adults.

Main effects	n	Sample size	SMD (95% CI)	I^2^	P-value
Overall effect	14	538	0.59 (0.33 to 0.85)	51%	< 0.001
Without outlier	13	482	0.49 (0.29 to 0.69)	11%	< 0.001
**Risk of bias**					
Low risk of bias^†^	1	24	0.30 (− 1.11 to 0.51)	–	–
Some concerns or high risk of bias	13	514	0.64 (0.39 to 0.89)	45%	< 0.001
**Population**					
Physically healthy	10	367	0.43 (0.21 to 0.64)	0%	< 0.001
Overweight/ Obese	2	73	0.79 (− 0.10 to 1.68)	67%	0.082
Moderate functional capacity^†^	1	42	0.72 (0.10 to 1.35)	–	–
Sarcopenic obesity^†^	1	56	1.69 (1.07 to 2.32)	–	–
**Intervention delivery**					
Supervised	13	492	0.61 (0.33 to 0.89)	54%	< 0.001
Unsupervised	–	–	−	–	–
**Intervention modality**					
Resistance exercise	10	353	0.64 (0.27 to 1.01)	63%	< 0.001
Resistance exercise + nutrition^†^	1	28	0.44 (− 0.32 to 1.20)	–	–
Aerobic exercise^†^	1	22	0.19 (− 0.65 to 1.03)	–	–
Aerobic exercise + nutrition^†^	1	21	0.57 (− 0.31 to 1.45)	–	–
Combined resistance and aerobic exercise^†^	1	49	0.33 (− 0.24 to 0.91)	–	–
Combined resistance and aerobic exercise + caloric restriction^†^	1	46	0.38 (− 0.21 to 0.97)	–	–
Multimodal exercise program^†^	1	42	0.72 (0.10 to 1.35)	–	–
**Assessment methods—knee extensors**					
1-RM/DXA	3	111	0.65 (0.26 to 1.05)	0%	0.001
1-RM/CT^†^	1	27	1.30 (0.45 to 2.14)	–	–
1-RM/US^†^	1	36	0.07 (− 0.58 to 0.72)	–	–
Isokinetic/DXA	5	195	0.48 (0.19 to 0.78)	0%	0.001
Isokinetic/CT	2	91	0.42 (− 0.16 to 0.99)	46%	0.155
Isometric/DXA^†^	1	56	1.69 (1.07 to 2.32)	–	–
Isometric/CT^†^	1	49	0.30 (− 0.27 to 0.87)	–	–
Isometric/BIA^†^	1	36	0.09 (− 0.58 to 0.76)	–	–
**Assessment methods—plantar flexors**					
30° sec^−1^/US^†^	1	24	− 0.46 (− 1.27 to 0.35)	–	–
Isometric/US^†^	1	24	0.00 (− 0.80 to 0.80)	–	–

^†^Insufficient data for analysis, BIA, Bioelectrical impedance analysis; CT, computed tomography; DXA, dual-energy X-ray absorptiometry; I^2^, indicator of between-study heterogeneity; n, number of studies; SMD, standardised mean difference; US, muscle ultrasound.

## Discussion

The present systematic review and meta-analysis examining the effects of exercise on morphological and neuromuscular MQ outcomes in older adults produced three important findings. First, there were significant positive effects of exercise on morphological MQ outcomes although the effects derived from different supervised exercise modalities were modest and not consistent across the subgroup analyses undertaken. Second, significant exercise effects were observed on neuromuscular MQ outcomes and these were mainly derived from supervised resistance exercise in physically healthy older adults. Third, there was no significant association between exercise intervention duration and morphological or neuromuscular MQ outcomes. Therefore, supervised exercise interventions significantly improved different measures of MQ regardless of exercise duration, although these effects were small-to-moderate and not supported across all population-, exercise-, and methods-related features.

Increased intramuscular and intermuscular adipose tissue and fibrous tissue accumulation are associated with aging and reduced oxidative capacity^39–41^, resulting in higher risk of metabolic syndrome and physical disabilities in older adults^11,42^. Although exercise has been widely suggested as an efficient treatment to counter or attenuate aging-related impairments in morphological MQ^8^, the present results indicate only a small effect derived from supervised exercise interventions on this outcome (ES =  ~ 0.3). Furthermore, results were not consistent across multiple subgroup analyses involving older adult populations, intervention-related characteristics, and assessment methods, precluding us observing potential moderators of exercise response on morphological MQ in older adults. The reasons for this may be related to the high relative risk of bias in most studies included in this review^17,18,20,23–25,29,30,36^ as well as the small number of exercise randomised controlled trials designed to directly investigate morphological MQ in this population^19,38^. In addition, the range of morphological MQ assessment characteristics may have affected the magnitude of exercise effects on this outcome since only specific measures from CT such as high muscle density and intermuscular adipose tissue of the thigh were improved by exercise, while exercise effects on other thigh measures and the calf muscles were not observed. For example, the location of the morphological MQ measurement, technology, and sensitivity to identify differences following exercise likely increases the heterogeneity among studies^43,44^. Therefore, these methodological issues may impede further analyses of exercise effects on MQ in older adults.

The ratio of muscle strength per muscle mass has been suggested as a more complex and complete index of muscle function in older adults^13,45^. In the present study, our findings are that exercise, mainly supervised resistance exercise is effective in improving neuromuscular MQ, although this was only significant in physically healthy older adults. This result is in accordance with previous research demonstrating more neural than morphological adaptations following short-term resistance training programs^16,24,33,37^. Therefore, despite expecting to observe greater effects in those at higher risk for disability (i.e., those presenting with low baseline levels), the paucity of studies examining exercise interventions in older adults with health issues impacting muscle precludes us observing such benefits in those most in need. In addition, most studies involved resistance training programs and strength increase with this exercise modality is expected following the principle of *specificity*^16^. However, this limited our ability for a more comprehensive analysis concerning different exercise modalities^18,26,27,35^ or the combination of exercise with nutrition interventions in older adults. For example, the combination of resistance, aerobic and balance exercises may counteract other age-related impairments (e.g., cardiorespiratory fitness, functional capacity, and body composition deficiencies), while the utilisation of protein supplementation has been associated with additional benefits in muscle strength and hypertrophy following exercise interventions^46^. Finally, inconsistency across different assessment methods for knee extensors and plantar flexors for strength and morphological features should be noted in our results as only knee extensor 1-RM and isokinetic muscle strength relative to DXA-derived measures were significantly enhanced by exercise^20,27,28,31,33,35,37^. Accordingly, methods to assess neuromuscular MQ outcomes must be carefully chosen and interpreted as this may determine the magnitude of adaptations observed and reported in older adults.

The association of exercise program duration with decreases in intramuscular and intermuscular fat or increases in muscle strength and size is not clear from the current literature^47,48^. In previous studies, greater morphological or neuromuscular adaptations were not observed following extended exercise program duration in older adults^47,48^, and this could be explained by the larger window for adaptations in untrained older persons during the initial 3 months of exercise compared to longer training periods (i.e., principle of *diminishing returns*)^49^. The present results are in accordance with these previous investigations^47,48^ indicating that MQ adaptations are mainly achieved during initial periods of training and maintained with longer training periods. Moreover, although we report no association between exercise program duration and greater effects on MQ, the required exercise volume and intensity to enhance MQ outcomes remains to be determined in older adults. In the present study, the high heterogeneity of exercise modalities, volume and intensity reported precluded the use of meta-regression analyses to examine if changes in MQ outcomes were dependent on higher exercise volumes or intensities. Therefore, although the necessary exercise dosage remains to be determined in this population, significant improvements in MQ outcomes might be achieved and maintained following short-term exercise programs, reducing the risk of metabolic disorders^5^ and functional impairments^3,4^ in older adults.

As far as we are aware, the present study is the first systematic review and meta-analysis to examine the exercise effects on MQ outcomes in older adults. The strengths of the present study are: (1) a large number of studies (n = 21) with up to ~ 1,000 participants; (2) the assessment of both neuromuscular and morphological derived MQ outcomes; and (3) a range of subgroup analyses based on different population characteristics, exercise modalities and delivery, and outcomes assessed. However, there are also some limitations which are worthy of comment. First, most studies included were of low quality because of concerns regarding the randomisation process and measurement of the outcomes. We attempted to use a subgroup analysis involving low risk of bias to minimise such bias; however, this was not possible given the small number of studies deemed *low risk*. Therefore, our results should be interpreted with caution because of the relatively low quality of studies included and small number of studies designed to directly investigate MQ as the main outcome. Second, all studies investigating MQ were supervised and mostly involved only resistance exercise programs and this may have limited our ability to detect the best intervention for morphological and neuromuscular MQ outcomes, or even if unsupervised exercise programs, a strategy of exercise delivery well-investigated recently^50^, may produce similar effects on these outcomes. Finally, there was high heterogeneity related to the older adult populations included and exercise components reported, impeding further analyses concerning the consistency of exercise effects across different population settings, and the exercise volume and intensity necessary to achieve improvements on MQ outcomes.

Regarding future research directions, despite promising findings from the current systematic review and meta-analysis examining the effects of exercise on MQ, well-designed trials are still required to determine the effectiveness of exercise on morphological MQ outcomes in older adults. For example, the effect of different exercise modalities or whether the combination of protein supplementation or other dietary strategies with exercise remains to be elucidated in various populations. Such questions are of great relevance given the importance of multidisciplinary strategies to counteract age-related impairments (e.g., cardiorespiratory fitness, functional capacity, and body composition deficiencies). Finally, additional studies are necessary to investigate the effects of exercise in older adults at increased risk of disability and the required exercise dosage to achieve meaningful benefits for MQ outcomes.

In summary, the present findings on the effects of exercise on muscle quality outcomes in older adults are promising. Our conclusions are that both morphological and neuromuscular MQ are improved by exercise interventions, although it was not consistent across different subgroup analyses involving different populations, methods, and exercise characteristics. Considering the evidence thus far, resistance exercise promotes greater effects on neuromuscular MQ in physically healthy older adults, and this appears to be achieved with relatively short-term programs, whereas a superior exercise mode was not observed for morphological MQ derived outcomes.

## Methods

### Study selection procedure

A systematic search was conducted in CINAHL, EMBASE, LILACS, PubMed, SciELO and Web of Science databases, while dissertations and theses (i.e., grey literature) were searched in MedNar, OpenGrey and OpenThesis databases, from inception to January 2021. The search strategy consisted of a combination of relevant keywords and controlled vocabulary that included the terms ‘age’, ‘resistance training’, ‘aerobic exercise’, ‘exercise’, ‘physical activity’, ‘muscle quality’, ‘intermuscular fat’, ‘intramuscular fat’, ‘specific tension’ and ‘randomised controlled trials’ (see in Appendix 1, Supplement Digital Content (SDC) 1, Search strategy). In addition, we also performed a manual search of the reference lists provided in the selected papers. All procedures were undertaken in accordance with the Preferred Reporting Items for Systematic Reviews and Meta-Analyses (PRISMA) statement^51,52^ and based on the minimum criteria established by the Cochrane Back Review Group (CBRG)^53^, with registration at the international prospective register of systematic reviews (PROSPERO identifier: CRD42021223794). In addition, the present systematic review complies with international guidelines and regulations as per the Declaration of Helsinki.

This review included randomised controlled trials evaluating the effects of supervised or unsupervised exercise programs combined or not with nutritional programs (e.g., protein supplementation, caloric restriction, or healthy diet) on morphological MQ outcomes, expressed as measures from CT (e.g., muscle density or intermuscular adipose tissue), pQCT (e.g., muscle density or intermuscular adipose tissue), MRI (e.g., lipid infiltration) or US (e.g., muscle echo intensity), and neuromuscular MQ outcomes expressed as the ratio of maximal muscle strength (e.g., derived from 1-RM (isotonic), isokinetic or isometric tests) per muscle mass parameter (e.g., muscle thickness, muscle cross-sectional area, muscle volume, lean mass, fat-free mass, or muscle mass) by MRI, CT, US, or dual-energy X-ray absorptiometry (DXA) in older adults (i.e., ≥ 60 years). The primary outcomes for this review were both morphological and neuromuscular MQ outcomes of the lower limb (e.g., thigh and calf muscles). The exclusion criteria were: (1) studies involving older adults with chronic conditions such as type II diabetes, cancer, chronic haemodialysis, or heart failure; (2) studies not including or reporting on the specific outcomes required for this review, or did not include sufficient information such as baseline and post-intervention assessment, or within- and between-group mean differences for analysis; (3) studies undertaking within-subject designs (i.e., legs randomised to different intervention programs or single-group studies); and (4) studies written in a language other than English, Portuguese or Spanish. In the search strategy, titles and abstracts were first independently evaluated following the eligibility criteria. When abstracts did not provide sufficient information, they were selected for full-text evaluation. Eligibility was assessed independently in duplicate (R. R. and P. L.) with differences resolved by consensus.

### Data extraction

Data extraction was performed via a standardised form. Demographic and methodological information were extracted from the included studies such as age, body mass index (BMI), number of participants randomised to study arms, exercise prescription characteristics that included duration, modality, frequency, intensity and volume, adverse events, and outcomes assessed. In addition, baseline, and post-intervention assessment, or within- and between-group mean difference from the outcomes of interest were extracted in their absolute units and for the longest period of the exercise intervention. When graphs were used instead of numerical data, the graphs were measured through the plots using a specific tool for data extraction (WebPlotDigitizer, San Francisco, CA)^54^.

### Risk of bias assessment

The risk of bias was evaluated according to the 2nd version of the Cochrane risk-of-bias tool for randomised trials (RoB 2) with each assessment focused at the outcome level^55^. The six-domain instrument includes: (1) randomisation process; (2) deviation from intended interventions; (3) missing outcome data; (4) measurement of the outcome; (5) selection of the reported result; and (6) overall bias. Overall risk of bias was expressed as “low risk of bias” if all domains were classified as low risk, “some concerns” if some concern was raised in at least one domain but not classified as at high risk in any other, or “high risk of bias” if at least one domain was classified as high risk, or multiple domains had some concerns^55^. The study quality assessment for all included studies were performed independently by two reviewers (R. R. and P. L.) with disagreements resolved by consensus.

### Data analysis

For the meta-analysis, the pooled effect estimates were obtained from the standardised mean difference (SMD) of baseline to the final assessment of the intervention versus control group. When studies did not provide dispersion values of change such as standard deviation (SD), standard errors or 95% confidence intervals (95% CI), the SD of the change was calculated by the square root of $\left({SD}_{Baseline}^{2}+ {SD}_{Post-intervention}^{2}\right)$, assuming a correlation of zero between the baseline and post-intervention assessment measures^56^. Furthermore, to avoid overestimating the weight of a study by entering it multiple times in the overall effect analyses, effects of different exercise groups were combined when reported/presented in the same study, as well as outcomes considered within the same outcome category (e.g., intermuscular adipose tissue and low muscle density)^57^. In outcomes where lower values indicate better than poorer results, the mean effect was multiplied by − 1 as recommended in the Cochrane Handbook^57^. Meta-analyses were conducted for overall studies, and subgroup analyses were provided for: (1) older adults subgroups (e.g., physically healthy, obese, mobility-limited, sarcopenic, frail); (2) exercise delivery modes (e.g., supervised vs. unsupervised exercise programs); (3) intervention modalities (e.g., resistance exercise, aerobic exercise, combined resistance and aerobic exercise, water-based exercise prescription, exercise plus nutritional supplementation); (4) outcomes assessment (e.g., muscle echo intensity, intermuscular adipose tissue); (5) thigh versus calf muscle outcomes (or knee extensors vs. plantar flexors); and (6) based on risk of bias assessment, when sufficient number of studies were available. Calculations were performed using a random-effects model with the DerSimonian & Laird method^58^. Statistical significance was assumed when the SMD effect was below an α level of P ≤ 0.05. Effect sizes (ES) were according to Cohen^59^ with values of 0.0 to < 0.5 indicating small, values of 0.51 to < 0.8 indicating medium, and values ≥ 0.8 indicating large effects. Statistical heterogeneity was assessed using the Cochran Q test^60^. A threshold P value of 0.1 as well as values greater than 50% in I^2^ were considered indicative of high heterogeneity^60^. We examined heterogeneity using sensitivity analysis by omitting one study at a time. Outliers were considered those studies in which the confidence intervals did not overlap the estimated pooled effect using the package ‘dmetar’ from R (function *find.outlier*; R Core Team, 2020). Publication bias was explored by contour-enhanced funnel plots and Egger’s test^61^ and, if necessary, trim-and-fill computation was used to estimate the effect of publication bias on the interpretation of results^60,62^. Analyses were conducted using the package ‘meta’ from R (R Core Team, 2020). Forest plots presented for the outcome measures are after sensitivity analysis procedure adjustments.

In addition, we tested the associations between exercise components (intervention duration, prescribed volume and peak intensity) and SMD effects if sufficient data were available. Using one or multiple variables at a time, we assessed whether exercise components influence the association of exercise with the main effects. Correlations were weighted by the inverse of the variance of each observation.

## Supplementary Information

Supplementary Information.
