# Supplementary material for: Exercise effects on muscle quality in older adults: a systematic review and meta-analysis
Source: Sci Rep. 2021 Oct 26;11:21085. doi: 10.1038/s41598-021-00600-3 (PMC8548567; doi:10.1038/s41598-021-00600-3)
Supplement: Supplementary file 1 — Supplementary Information. [file 41598_2021_600_MOESM1_ESM.docx]

**Supplemental digital content**

Radaelli et al., **Exercise effects on muscle quality in older adults: A systematic review and meta-analysis**

**Supplement Digital Content Appendix S1.** Search strategy

**Supplement Digital Content Table S1.** Study characteristics: experimental design and sample size, exercise prescription and outcomes assessed.

**Supplement Digital Content Figure S1.** Individual risk of bias assessment at outcome level for A) morphological muscle quality and B) neuromuscular muscle quality outcomes. Green circles, low risk; yellow circles, some concerns; red circles, high risk of bias.

**Supplement Digital Content Figure S2.** Contour-enhanced funnel plot for A) morphological muscle quality and B) neuromuscular muscle quality outcomes.

**Appendix 1.** Search strategy

*Search strategy for PubMed*

(Aged [Mesh] OR Aged [title/abstract] OR Aging [title/abstract] OR Ageing [title/abstract] OR Old [title/abstract] OR Older [title/abstract] OR Elderly [title/abstract] OR Senior [title/abstract] OR Geriatric [title/abstract] OR Elderly, Frail [title/abstract] OR Frail Elders [title/abstract] OR Elder, Frail [title/abstract] OR Elders, Frail [title/abstract] OR Frail Elder [title/abstract] OR Functionally-Impaired Elderly [title/abstract] OR Elderly, Functionally-Impaired [title/abstract] OR Functionally Impaired Elderly [title/abstract] OR Frail Older Adults [title/abstract] OR Adult, Frail Older [title/abstract] OR Adults, Frail Older [title/abstract] OR Frail Older Adult [title/abstract] OR Older Adult, Frail [title/abstract] OR Older Adults, Frail [title/abstract]) AND (“Resistance Exercise” [title/abstract] OR “Resistance Training” [title/abstract] OR Training, Resistance [title/abstract] OR “Strength Exercise” OR “Strength Training” [title/abstract] OR Training, Strength [title/abstract] OR Weight-Lifting Strengthening Program [title/abstract] OR Strengthening Program, Weight-Lifting [title/abstract] OR Strengthening Programs, Weight-Lifting [title/abstract] OR Weight Lifting Strengthening Program [title/abstract] OR Weight-Lifting Strengthening Programs [title/abstract] OR Weight-Lifting Exercise Program [title/abstract] OR Exercise Program, Weight-Lifting [title/abstract] OR Exercise Programs, Weight-Lifting [title/abstract] OR Weight Lifting Exercise Program [title/abstract] OR Weight-Lifting Exercise Programs [title/abstract] OR Weight-Bearing Strengthening Program [title/abstract] OR Strengthening Program, Weight-Bearing [title/abstract] OR Strengthening Programs, Weight-Bearing [title/abstract] OR Weight Bearing Strengthening Program [title/abstract] OR Weight-Bearing Strengthening Programs [title/abstract] OR Weight-Bearing Exercise Program [title/abstract] OR Exercise Program, Weight-Bearing [title/abstract] OR Exercise Programs, Weight-Bearing [title/abstract] OR Weight Bearing Exercise Program [title/abstract] OR Weight-Bearing Exercise Programs [title/abstract] OR Exercises [title/abstract] OR Exercise, Physical [title/abstract] OR Exercises, Physical [title/abstract] OR Physical Exercise [title/abstract] OR Physical Exercises [title/abstract] OR Physical Activity [title/abstract] OR Activities, Physical [title/abstract] OR Activity, Physical [title/abstract] OR Physical Activities [title/abstract] OR Circuit Based Exercise [title/abstract] OR Circuit-Based Exercises [title/abstract] OR Exercise, Circuit-Based [title/abstract] OR Exercises, Circuit-Based [title/abstract] OR Circuit Training [title/abstract] OR Training, Circuit [title/abstract] OR Exercise, Isometric [title/abstract] OR Exercises, Isometric [title/abstract] OR Isometric Exercises [title/abstract] OR Isometric Exercise [title/abstract] OR Exercise, Aerobic [title/abstract] OR “Aerobic Exercise” [title/abstract] OR Aerobic Exercises [title/abstract] OR Exercises, Aerobic [title/abstract] OR Exercise Training [title/abstract] OR Exercise Trainings [title/abstract] OR Training, Exercise [title/abstract] OR Trainings, Exercise [title/abstract] OR High Intensity Interval Training [title/abstract] OR High-Intensity Interval Trainings [title/abstract] OR Interval Training, High-Intensity [title/abstract] OR Interval Trainings, High-Intensity [title/abstract] OR Training, High-Intensity Interval [title/abstract] OR Trainings, High-Intensity Interval [title/abstract] OR High-Intensity Intermittent Exercise [title/abstract] OR Exercise, High-Intensity Intermittent [title/abstract] OR Exercises, High-Intensity Intermittent [title/abstract] OR High-Intensity Intermittent Exercises [title/abstract]) AND (“muscle quality” [title/abstract] OR “specific tension” [title/abstract] OR “echo intensity” [title/abstract] OR echogenicity [title/abstract] OR “intermuscular adipose tissue” [title/abstract] OR “intermuscular fat” [title/abstract] OR “intramuscular adipose tissue” [title/abstract] OR “intramuscular fat” [title/abstract] OR “muscle attenuation” [title/abstract] OR “muscle density” [title/abstract] OR “radiological density” [title/abstract] OR “muscle composition” [title/abstract] OR “muscle fat infiltration” [title/abstract] OR “myosteatosis” [title/abstract]) AND (“clinical trial” [title/abstract] OR “randomized controlled trial”[title/abstract] OR “controlled clinical trial”[title/abstract] OR “randomized”[title/abstract] OR “randomly”[title/abstract] OR “groups” [title/abstract] OR “trial” [title/abstract])

*Search strategy for CINAHL, SportDiscus and Web of Science*

(Aged OR Aged OR Aging OR Ageing OR Old OR Older OR Elderly OR Senior OR Geriatric OR Elderly, Frail OR Frail Elders OR Elder, Frail OR Elders, Frail OR Frail Elder OR Functionally-Impaired Elderly OR Elderly, Functionally-Impaired OR Functionally Impaired Elderly OR Frail Older Adults OR Adult, Frail Older OR Adults, Frail Older OR Frail Older Adult OR Older Adult, Frail OR Older Adults, Frail) AND (“Resistance Exercise” OR “Resistance Training” OR Training, Resistance OR “Strength Exercise” OR “Strength Training” OR Training, Strength OR Weight-Lifting Strengthening Program OR Strengthening Program, Weight-Lifting OR Strengthening Programs, Weight-Lifting OR Weight Lifting Strengthening Program OR Weight-Lifting Strengthening Programs OR Weight-Lifting Exercise Program OR Exercise Program, Weight-Lifting OR Exercise Programs, Weight-Lifting OR Weight Lifting Exercise Program OR Weight-Lifting Exercise Programs OR Weight-Bearing Strengthening Program OR Strengthening Program, Weight-Bearing OR Strengthening Programs, Weight-Bearing OR Weight Bearing Strengthening Program OR Weight-Bearing Strengthening Programs OR Weight-Bearing Exercise Program OR Exercise Program, Weight-Bearing OR Exercise Programs, Weight-Bearing OR Weight Bearing Exercise Program OR Weight-Bearing Exercise Programs OR Exercises OR Exercise, Physical OR Exercises, Physical OR Physical Exercise OR Physical Exercises OR Physical Activity OR Activities, Physical OR Activity, Physical OR Physical Activities OR Circuit Based Exercise OR Circuit-Based Exercises OR Exercise, Circuit-Based OR Exercises, Circuit-Based OR Circuit Training OR Training, Circuit OR Exercise, Isometric OR Exercises, Isometric OR Isometric Exercises OR Isometric Exercise OR Exercise, Aerobic OR “Aerobic Exercise” OR Aerobic Exercises OR Exercises, Aerobic OR Exercise Training OR Exercise Trainings OR Training, Exercise OR Trainings, Exercise OR High Intensity Interval Training OR High-Intensity Interval Trainings OR Interval Training, High-Intensity OR Interval Trainings, High-Intensity OR Training, High-Intensity Interval OR Trainings, High-Intensity Interval OR High-Intensity Intermittent Exercise OR Exercise, High-Intensity Intermittent OR Exercises, High-Intensity Intermittent OR High-Intensity Intermittent Exercises) AND (“muscle quality” OR “specific tension” OR “echo intensity” OR echogenicity OR “intermuscular adipose tissue” OR “intermuscular fat” OR “intramuscular adipose tissue” OR “intramuscular fat” OR “muscle attenuation” OR “muscle density” OR “muscle composition” OR “muscle fat infiltration” OR “myosteatosis”) AND (“clinical trial” OR “randomized controlled trial” OR “controlled clinical trial” OR “randomized” OR “randomly” OR “groups” OR “trial”)

*Search strategy for EMBASE*

(aged OR aging OR 'elderly care' OR 'older adults' OR geriatric OR 'frail elderly') AND ('resistance training' OR 'aerobic training' OR exercise OR 'isometric exercise' OR 'high intensity interval training') AND ('muscle quality' OR 'echo intensity' OR echogenicity OR 'intermuscular adipose tissue' OR 'intermuscular fat' OR 'intramuscular adipose tissue' OR 'intramuscular fat' OR 'muscle attenuation' OR 'muscle density' OR 'muscle fat infiltration' OR myosteatosis) AND ('clinical trial' OR 'randomized controlled trial' OR 'controlled clinical trial' OR trial OR group)

*Search strategy for LILACS and Scielo*

(idoso OR mayor OR anciano OR Viejo) AND (“treinamento de força” OR “treinamento resistido” OR musculação OR “entrenamiento de Resistencia” OR “entrenamiento de fuerza” OR “treinamento aerobico” OR “entrenamiento aerobico” OR exercicio OR ejercicio OR entrenamiento OR “exercicio isometrico” OR “ejercicio isometrico” OR “treinamento intervalado de alta intensidade” OR “Entrenamiento de intervalos de alta intensidad”) AND (“qualidade muscular” OR “calidad muscular” OR “tensão especifica” OR “tensión específica” OR “eco intensidade” OR “intensidad del eco” OR ecogenicidade OR ecogenicidad OR “tecido adiposo intermuscular” OR “tejido adiposo intermuscular” OR “gordura intermuscular” OR “grasa intermuscular” OR “tecido adipose intramuscular” OR “tejido adiposo intramuscular” OR “gordura intramuscular” OR “grasa intramuscular” OR “atenuação muscular” OR “atenuación muscular” OR “densidade muscular” OR “densidad muscular” OR “miosteatose” OR “miosteatosis”)

*Search strategy for Open Thesis, Open Grey and MedNar*

(older OR elderly OR idoso OR mayor OR anciano OR Viejo) AND (“resistance training” OR “strength training” OR “treinamento de força” OR “treinamento resistido” OR musculação OR “entrenamiento de resistencia” OR “entrenamiento de fuerza” OR “aerobic training” OR “treinamento aerobico” OR “entrenamiento aerobico” OR exercise OR exercicio OR ejercicio OR entrenamiento OR “isometric exercise” OR “treinamento isometrico” OR “ejercicio isometrico” OR “high intensity interval training” OR “treinamento intervalado de alta intensidade” OR “Entrenamiento de intervalos de alta intensidad”) AND (“muscle quality” OR “qualidade muscular” OR “calidad muscular” OR “specific tension” OR “tensão especifica” OR “tensión específica” OR “echo intensity” OR “eco intensidade” OR “intensidad del eco” OR echogenicity OR ecogenicidade OR ecogenicidad OR “'intermuscular adipose tissue” OR “tecido adiposo intermuscular” OR “tejido adiposo intermuscular” OR “intermuscular fat” OR “gordura intermuscular” OR “grasa intermuscular” OR “intramuscular adipose tissue” OR “tecido adipose intramuscular” OR “tejido adiposo intramuscular” OR “intramuscular fat” OR “gordura intramuscular” OR “grasa intramuscular” OR “muscle attenuation” OR “atenuação muscular” OR “atenuación muscular” OR “muscle density” OR “densidade muscular” OR “densidad muscular” OR “myosteatosis” OR “miosteatose” OR “miosteatosis”)

**Table S1.** Study characteristics: experimental design and sample size, exercise prescription and outcomes assessed.

| **Author, year** | **Participants’ characteristics** | **Experimental design** | **Exercise intervention prescription** | **Adverse events** | **Outcomes** |
| --- | --- | --- | --- | --- | --- |
| Sipila & Suominen, 1995 (52) | Physically healthy older women  Age: from 76 to 78 yrs  BMI: from 26.3 to 27.4 kg.m^-2^ | Resistance exercise  vs.  Aerobic exercise  vs.  Non-active control group | **Resistance exercise**  n= 16, 2 sessions per week for 18 weeks performing a supervised program undertaking 3-4 sets of 8-10 reps at 60-75% of 1-RM;  **Aerobic exercise**  n= 15, 2 sessions per week for 18 weeks performing a supervised program undertaking ~1 hour at an intensity of 50-80% HR. | Two participants in the resistance exercise group experienced mild muscle soreness in the calf area. | Knee flexor and extensor muscle density (CT) |
| Goodpaster et al., 2008 (20) | Older adults with moderate limited functional capacity  Age: mean of 77.1 yrs  BMI: mean of 30.5 kg.m^-2^ | Multimodal exercise program  vs.  Health education control group | **Multimodal exercise program**  n=22, 1-3 sessions per week for 48 weeks performing a supervised program undertaking 40-60 min of walking as the primary mode of exercise, resistance exercise, flexibility and balance exercises. Sessions were supplemented by home-based exercises from weeks 9 to 48. | No adverse events | Isokinetic concentric knee extension to knee extensors muscle cross-sectional area (CT) ratio;  Thigh muscle density and intermuscular adipose tissue (CT) |
| Avila et al., 2010 (2) | Overweight and obese older adults  Age: mean of 66.7 yrs  BMI: mean of 31.7 kg.m^-2^ | Resistance exercise  vs.  Dietary education control | **Resistance exercise**  n=15, 3 sessions per week for 10 weeks performing a supervised program undertaking 4 sets of 8-12 RM. In addition, the resistance exercise group also received 30-min dietary educational classes. | One participant in the resistance exercise group experienced a minor hip extensor muscle strain. | Thigh intermuscular adipose tissue (CT);  Knee extensor 1-RM to knee extensors muscle cross-sectional area (CT) ratio |
| Bergamin et al., 2013 (3) | Physically healthy older adults  Age: mean of 71.7 yrs  BMI: mean of 25.5 kg.m^-2^ | Aquatic resistance exercise  vs.  Resistance exercise  vs  Non-active control group | **Aquatic group**  n=17, 2 sessions per week for 24 weeks performing a supervised program undertaking five exercises for upper- and lower-body at 13 and 16 Borg scale  **Resistance exercise**  n=17, 2 sessions per week for 24 weeks performing a supervised program involving five exercises for upper- and lower-body at 13 and 16 Borg scale. | No adverse events | Lower leg muscle density  (pQCT) |
| Kennis et al., 2013 (27) | Physically healthy older men  Age: mean of 68.0 yrs  BMI: Not reported | Combined resistance and aerobic exercise  vs.  Non-active control group | **Combined resistance and aerobic exercise**  n=20, 3 sessions per week for 48 weeks performing a supervised program involving walking, cycling or stepping at 75% and 85% of heart rate reserve, and undertaking 1-2 sets of 8-15 RM of resistance exercise plus balance exercise. | NR | Isometric and isokinetic concentric knee extension to knee extensor muscle volume (CT) ratio |
| Miller & Robinson, 2013 (34) | Overweight and obese older women  Age: mean of 67.8 ± 1.3 yrs  BMI: mean of 34.9 ± 0.7 kg.m^-2^ | Combined resistance and aerobic exercise plus caloric restriction  vs.  Health education control group | **Combined resistance and aerobic exercise plus caloric restriction** n=26, 3 sessions per weeks for 24 weeks performing a program undertaking combined resistance and aerobic exercise for 60 min per session. Participants also received a nutrition education and lifestyle behaviour modification component targeting 10% of weight loss during the program. | NR | Isokinetic concentric knee extension to leg lean soft tissue (DXA) ratio |
| Cadore et al., 2014 (4) | Frail older adults  Age: mean of 91.9 ± 4.1 yrs  BMI: Not reported | Multicomponent exercise program  vs.  Stretching | **Multimodal exercise program**  n=11, 2 sessions per week for 12 weeks of performing a supervised program undertaking 8-10 reps at 40–60 % of 1-RM of resistance exercise combined with balance and gait exercises, and functional exercises. | No adverse events | Thing muscle density (CT) |
| Fragala et al., 2014 (17) | Physically healthy older adults  Age: mean of 70.0 yrs  BMI: mean of 28.0 kg.m^-2^ | Resistance exercise  vs.  Non-active control group | **Resistance exercise**  n=12, 2 sessions per week for 24 weeks performing a supervised program undertaking 3 sets of 8-15 reps at ∼70 to 85 % of 1-RM. | NR | Predicted knee extension strength to lean quadriceps muscle mass (DXA) ratio |
| Pinto et al., 2014 (45) | Physically healthy older women  Age: mean of 66.0 ± 8 yrs  BMI: not reported | Resistance exercise  vs.  Non-active control group | **Resistance exercise**  n=19, 2 sessions per week for six weeks  performing a supervised program undertaking 2-3 sets of 12-20 RM. | NR | Knee extension 1-RM to quadriceps muscle thickness (US) ratio |
| Scanlon et al., 2014 (51) | Physically healthy older adults  Age: mean of 70.6 yrs  BMI: mean of 28.0 kg.m^2^ | Resistance exercise  vs.  Non-active control group | **Resistance exercise**  n=13, 2 sessions per week for six weeks performing a supervised program undertaking 2-4 sets of 8-12 reps at ∼70 to 85 % of 1-RM. | NR | Rectus Femoris and Vastus Lateralis muscle echo intensity (US);  Knee extension 1-RM to lean thigh muscle mass (DXA) ratio |
| Wilhelm et al., 2014 (60) | Physically healthy older men  Age: mean of 65.2 yrs  BMI: mean of 25.8 kg.m^2^ | Combined resistance and aerobic exercise  vs.  Non-active control group | **Combined resistance and aerobic exercise**  n=30, 2 sessions per week for 12 weeks performing a supervised program undertaking 2-3 sets of 8-18RM of resistance exercise and 20-40 min at 85-95% HRVT2 of aerobic exercise. | NR | Rectus Femoris muscle echo intensity (US) |
| Ikenaga et al., 2017 (26) | Physically healthy older adults  Age: mean of 70.9 yrs  BMI: mean of 23.0 kg.m^2^ | Aerobic exercise  vs  Non-active control group | **Aerobic exercise**  n=37, 1 session per week for 12 weeks performing a supervised program undertaking 180 min of slow-jogging per week. | One participant in the exercise group dropped out because of worsening knee osteoarthritis pain. | Thigh muscle density and intermuscular adipose tissue (CT) |
| Oh et al., 2017 (39) | Physically healthy older women  Age: mean of 74.2 yrs  BMI: mean of 24.9 kg.m^2^ | Resistance exercise  vs.  Stretching | **Resistance exercise**  n=19, 1 session per week for 18 weeks performing a supervised program involving  health education and undertaking two sessions per week of resistance exercise comprising 2-3 sets of 10-20 RM using elastic bands. | Four participants in the exercise group developed existing soft tissue injuries in the knee regions and dropped out of the study. | Isometric and isokinetic concentric knee extension to leg muscle mass (DXA) ratio |
| Liao et al., 2018 (29) | Older women with sarcopenic obesity  Age: mean of 67.4 yrs  BMI: mean of 28.2 kg.m^2^ | Resistance exercise  vs.  Non-active control group | **Resistance exercise**  n=33, 3 sessions per week for 12 weeks  performing a supervised program undertaking 3 sets of 10 repetitions at 13 RPE using elastic bands. | No adverse events | Isometric knee extension to leg lean mass (DXA) ratio |
| Markofski et al., 2018 (33) | Physically healthy older adults  Age: mean of 72.0 ± 1.0  BMI: mean of 26.9 kg.m^2^ | Aerobic exercise + Supplementation  vs.  Aerobic exercise + Placebo  vs.  Placebo | **Aerobic exercise + Supplementation**  n=14, 3 sessions per week for 24 weeks  performing a supervised program undertaking 45 min of progressive aerobic exercise at 70% of heart rate reserve. In addition, participants received a supplementation of essential amino acids.  **Aerobic exercise + Placebo**  n =13, 3 sessions per week for 24 weeks  performing a supervised program undertaking 45 min of progressive aerobic exercise at 70% of heart rate reserve. In addition, participants received a placebo supplementation. | No adverse events | Isokinetic concentric knee extension to whole leg lean mass (DXA) ratio |
| Strasser et al., 2018 (57) | Physically healthy older adults  Age: mean 82.6 yrs  BMI: mean of 28.6 kg.m^2^ | Resistance exercise + Supplementation  vs.  Resistance exercise  vs.  Cognitive and coordinative tasks | **Resistance exercise + Supplementation**  n=21, 2 sessions per week for 24 weeks performing a supervised program undertaking resistance exercise using elastic bands. In addition, participants received a nutrition supplement drink containing 20.7 g of protein, 9.3 g of carbohydrates, 3 g of fat, vitamins and minerals.  **Resistance exercise**  n=16, 2 sessions per week for 24 weeks performing a supervised program undertaking resistance exercise using elastic bands. | No adverse events | Isokinetic concentric knee extension to lean leg mass (DXA) ratio |
| Coelho-Júnior et al., 2019 (6) | Physically healthy older women  Age: mean of 66.8 yrs  BMI: mean of 28.3 kg.m^2^ | Resistance exercise  vs.  Non-active control group | **Resistance exercise**  n=22, 2 sessions per week for 18 weeks performing a supervised program undertaking 3 sets of 8-10 reps at 5-6 RPE using elastic bands. | NR | Isometric knee extension to lower extremity muscle mass (BIA) ratio |
| Yamada et al., 2019 (62) | Older adults with sarcopenia and dynapenia  Age: mean of 84.0 yrs  BMI: mean of 21.9 kg.m^2^ | Resistance exercise + Supplementation  vs.  Resistance exercise  vs.  Non-active control group | **Resistance exercise + supplementation**  n=28, 2 sessions per week for 12 weeks performing a supervised program undertaking 30 min of bodyweight resistance exercises. In addition, participants received protein and vitamin D supplementation every day.  **Resistance exercise**  n=28, 2 sessions per week for 12 weeks performing a supervised program undertaking 30 min of bodyweight resistance exercises. | No adverse events | Rectus Femoris and Vastus Intermedius muscle echo intensity (US) |
| Cunha et al., 2020 (9) | Physically healthy older women  Age: mean of 69.0 yrs  BMI: mean of 27.0 kg.m^2^ | Resistance exercise  vs.  Non-active control group | **Resistance exercise**  n=41, 3 sessions per week for 12 weeks performing a supervised program undertaking 1 or 3 sets of 10-15 RM. | NR | Knee extension 1-RM to lower-limb lean soft-tissue (DXA) ratio |
| Lopez et al., 2020 (31) | Physically healthy older women  Age: mean of 66.3 ± 5.8 yrs  BMI: mean of 25.3 kg.m^2^ | Resistance exercise  vs  Stretching and walk | **Resistance exercise**  n=12, 2 sessions per week for 8 weeks performing a supervised program undertaking 3 sets of 6-12 reps at 8-15 RM. | NR | Gastrocnemius and Soleus muscle echo intensity (US);  Isometric and isokinetic concentric plantar flexors to plantar flexors muscle thickness (US) ratio |
| Minett et al., 2020 (35) | Physically healthy older adults  Age: mean of 76.4 yrs  BMI: NR | Multicomponent exercise program  vs  Walk | **Multicomponent exercise program**  n=44, 2 sessions per week for 12 weeks performing a supervised program undertaking 30 min of walk in addition to exercises targeting muscle strength and balance | NR | Calf muscle density and intramuscular adipose tissue (pQCT) |

1-RM, One-repetition maximum; BIA, bioelectrical impedance analysis, BMI, body mass index; CT, computerised tomography; DXA, dual-energy X-ray absorptiometry; HR, heart rate; HRVT, heart rate variability threshold; n, number of participants; NR, not reported; pQCT, peripheral quantitative computerised tomography; RM, repetition maximum; RPE, rate perceived exertion; US, ultrasound.


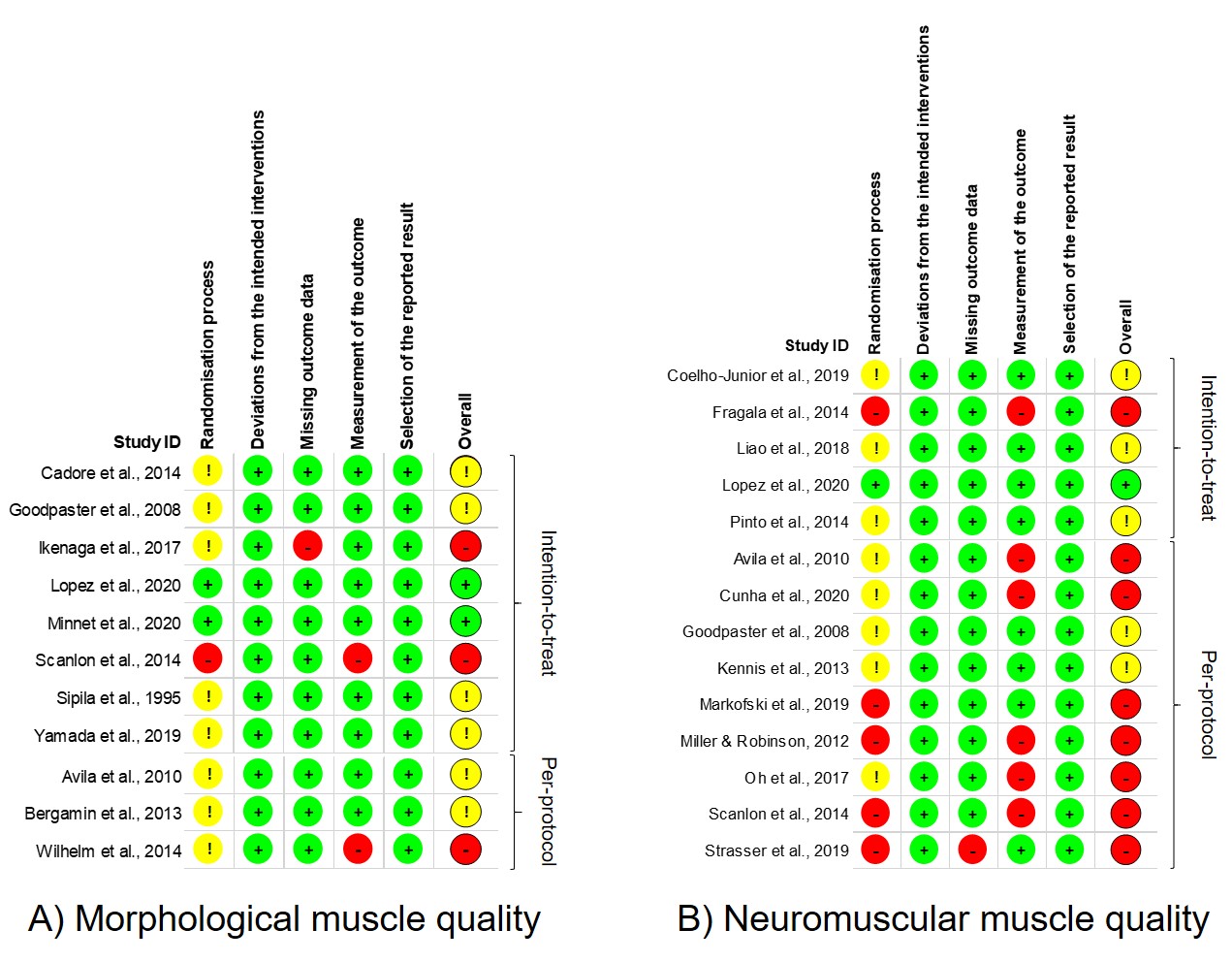


**Figure S1.** Individual risk of bias assessment at outcome level for A) morphological muscle quality and B) neuromuscular muscle quality outcomes. Green circles, low risk; yellow circles, some concerns; red circles, high risk of bias.


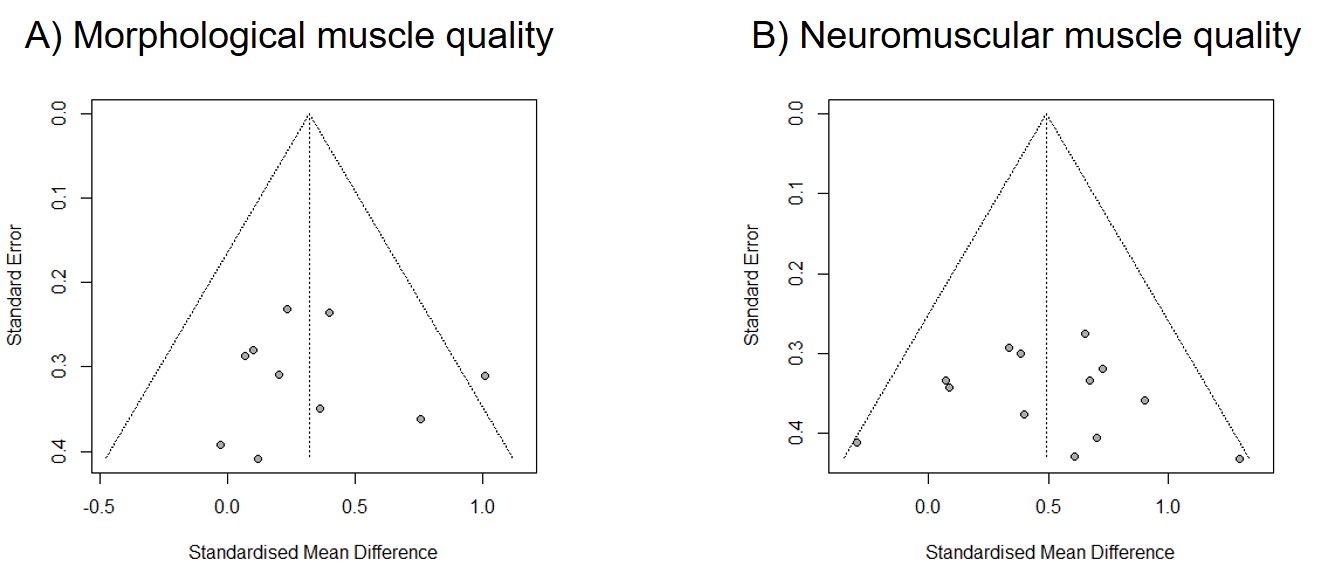


**Figure S2.** Contour-enhanced funnel plot for A) morphological muscle quality and B) neuromuscular muscle quality outcomes.
